# Impact of *UGT1A1* gene polymorphisms on plasma dolutegravir trough concentrations and neuropsychiatric adverse events in Japanese individuals infected with HIV-1

**DOI:** 10.1186/s12879-017-2717-x

**Published:** 2017-09-16

**Authors:** Hiroki Yagura, Dai Watanabe, Hiroyuki Kushida, Kosuke Tomishima, Hiroaki Togami, Atsushi Hirano, Masaaki Takahashi, Kazuyuki Hirota, Motoko Ikuma, Daisuke Kasai, Yasuharu Nishida, Munehiro Yoshino, Kunio Yamazaki, Tomoko Uehira, Takuma Shirasaka

**Affiliations:** 1 Department of Pharmacy, National Hospital Organization Osaka National Hospital, 2-1-14, Hoenzaka, Chuo-ku, Osaka City, 540-0006 Japan; 2 AIDS Medical Center, National Hospital Organization Osaka National Hospital, 2-1-14, Hoenzaka, Chuo-ku, Osaka City, 540-0006 Japan; 3 Department of Pharmacy, National Hospital Organization Nagoya Medical Center, 4-1-1, Sannomaru, Naka-ku, Nagoya City, Aichi 460-0001 Japan; 4 Department of Pharmacy, National Hospital Organization Suzuka Hospital, 3-2-1 Kasado, Suzuka, Mie 513-8501 Japan; 5 Department of Pharmacy, National Hospital Organization Utano Hospital, Narutaki, Ondoyama-cho 8, Ukyo-ku, Kyoto, Japan

**Keywords:** Dolutegravir, Plasma trough concentration, *UGT1A1* gene polymorphism, Neuropsychiatric adverse events

## Abstract

**Background:**

Dolutegravir (DTG) is metabolized mainly by uridine diphosphate (UDP)-glucuronosyltransferase 1A1 (UGT1A1), and partly by cytochrome P450 3A (CYP3A). Therefore, we focused on *UGT1A1* gene polymorphisms (*6 and *28) in Japanese individuals infected with human immunodeficiency virus (HIV)-1 to examine the relationship between their plasma trough concentration of DTG and gene polymorphisms. Recently, neuropsychiatric adverse events (NP-AEs) after the use of DTG have become a concern, so the association between *UGT1A1* gene polymorphisms and selected NP-AEs was also investigated.

**Methods:**

The study subjects were 107 Japanese patients with HIV-1 infections who were receiving DTG. Five symptoms (dizziness, headache, insomnia, restlessness, and anxiety) were selected as NP-AEs. The subjects were classified by their *UGT1A1* gene polymorphisms for the group comparison of DTG trough concentration and the presence or absence of NP-AEs.

**Results:**

The subjects consisted of eight (7%) *6 homozygotes, three (3%) *28 homozygotes, four (4%) for *6/*28 compound heterozygotes, 23 (21%) *6 heterozygotes, 18 (17%) *28 heterozygotes, and 51 (48%) patients carrying the normal allele. The plasma DTG trough concentration of the *6 homozygous patients was significantly higher than that of the patients carrying the normal allele (median, 1.43 and 0.82 μg/mL, respectively, *p* = 0.0054). The *6 and *28 heterozygous patients also showed significantly higher values than those shown by patients with the normal allele. Multivariate analysis revealed that carrying one or two UGT1A1*6 gene polymorphisms, one UGT1A1*28 polymorphism, and age of < 40 years were independent factors associated with high DTG trough concentrations. The median DTG trough concentration was significantly higher in the patients with NP-AEs (1.31 μg/mL) than in those without NP-AEs (1.01 μg/mL). Consistent with these results, subjects carrying UGT1A1*6, UGT1A1*28, or both alleles showed a higher cumulative incidence of having selected NP-AEs than those carrying the normal alleles (*p* = 0.0454).

**Conclusion:**

In addition to younger age, carrying UGT1A1*6 and/or UGT1A1*28 was demonstrated to be a factor associated with high DTG trough concentrations. Our results also suggest a relationship between plasma DTG trough concentrations and NP-AEs, and that carrying UGT1A1*6 and/or UGT1A1*28 alleles might be a risk factor for NP-AEs.

## Background

Dolutegravir (DTG) is a second-generation human immunodeficiency virus (HIV)-1 integrase inhibitor. In patients without drug-resistant mutations related to integrase inhibitors, once daily administration of DTG shows a good virological effect [1–4]. Some clinical trials have demonstrated the virological superiority of DTG in comparison with that of other agents [1–4]. Therefore, major guidelines list DTG as a recommended key drug in the initial therapy of HIV infection [5, 6]. DTG is metabolized mainly by uridine diphosphate (UDP)-glucuronosyltransferase 1A1 (UGT1A1) and partly by cytochrome P450 (CYP) 3A. DTG does not have inhibitory or inducing effects on CYP and UGT, and therefore, has a lower propensity for drug-drug interactions compared to other antiretroviral drugs [7].

A previous population pharmacokinetic analysis has shown a negative correlation between total bilirubin and DTG’s apparent clearance, which has been attributed to the competition of bilirubin and DTG for UGT1A1. Hence, it is likely that changes in UGT1A1 enzymatic activity and its protein level might affect plasma DTG concentrations [8].

The *UGT1A1* gene has several polymorphisms. The promoter region of UGT1A1 normally has six TA repeats in the TATA box, whereas the number is increased to seven in UGT1A1*28, which lowers the mRNA and protein expression levels of UGT1A1 [9]. In addition, the single nucleotide polymorphism in exon 1 of UGT1A1*6 reduces UGT1A1 activity. The frequency of the *UGT1A1* gene polymorphisms has been demonstrated to vary depending on race. UGT1A1*28 is relatively common, whereas UGT1A1*6 is rarely found in Caucasian and African American populations [10]. On the other hand, *UGT1A1* *6 frequency is high in Asian populations, including Japanese [11, 12].

In a previous study conducted in Caucasian and African American populations, the area under the DTG plasma concentration-time curve (AUC) and the maximum DTG plasma concentration (C_max_) of patients homozygous for UGT1A1*28 were approximately 1.3–1.4 times higher than those of patients carrying normal alleles [13]. However, the effect of the UGT1A1*6 allele was not fully analyzed because few subjects carrying that allele were found in that study. Therefore, we examined the effects of carrying the UGT1A1*6, *28, or both gene polymorphisms on plasma trough concentrations of DTG in Japanese individuals with HIV-1 infections.

Recently, neuropsychiatric adverse events (NP-AEs) after the use of DTG have become a concern [14–20]. Although a low discontinuation rate of DTG has been reported in phase III clinical trials [1–4], NP-AEs are known to be a major cause of DTG discontinuation in real-life setting. Therefore, the association between *UGT1A1* gene polymorphisms and selected NP-AEs was also investigated.

## Methods

### Patients

This study included Japanese patients infected with HIV-1 who visited the National Hospital Organization Osaka National Hospital, who were 20 years old or older, and who received anti-HIV therapy in combination with DTG. Written consent was acquired prior to sample collection and testing. The subjects were limited to those taking DTG in the morning, because of the time limitations of our institution on the collection and processing of patient samples. We interviewed them about their medication adherence to exclude subjects with poor adherence. Subjects concomitantly using a potent inhibitor of UGT1A1 (atazanavir), potent inducers of UGT1A1, CYP3A, or both (rifampicin, carbamazepine, phenytoin, and phenobarbital), potent inhibitors of CYP3A (itraconazole, cobicistat, and ritonavir), and any other drugs that could affect plasma DTG concentrations (aluminum- or magnesium-containing antacids or both) were excluded.

### UGT1A1 Genotyping and plasma trough concentration of DTG

Saliva samples were collected from the patients, absorbed with a filter paper, and subsequently dried. A portion of the filter paper containing the saliva sample was cut and used as a template in the polymerase chain reaction (PCR) used to determine the presence or absence of gene polymorphisms using a previously reported method [21].

Blood samples were collected after oral administration of DTG for 10 days or longer. Whole blood samples (5 mL) were collected in test tubes containing heparin sodium 22–26 h after an oral administration of DTG. The blood samples were centrifuged at 1200×*g* for 10 min to isolate 2 mL plasma, which was stored in a freezer at −80°C until assayed. We measured the DTG plasma concentration using mass spectrometry based on our previously developed assay for raltegravir (RAL) [22], an integrase inhibitor. DTG was detected at a mass-to-charge ratio (m/z) of 420 using the MassLynx analysis software version 4.0 (Waters Corp., Milford, MA).

### Examination of the relationship of UGT1A1 genotype and DTG plasma trough concentration to NP-AEs

Five symptoms (dizziness, headache, insomnia, restlessness, and anxiety) were selected as NP-AEs. The information on NP-AEs was obtained upon patient entry into this study, and the subjects were followed-up until March 2017 or the discontinuation of DTG. NP-AEs, which had developed after starting DTG but had ceased by initiation of the study, were included in the analyses. Plasma DTG trough concentrations were compared between the subjects with or without NP-AEs.

### Statistical analysis

For group comparisons, the Wilcoxon rank-sum test was used. If a statistically significant result was obtained for multiple group comparison, Steel’s test was used as the post-hoc test. To search for factors associated with trough concentrations of DTG, multivariate analysis using logistic regression was conducted. The subjects were classified into two groups using the median trough DTG concentration (1.06 μg/mL) as a cut-off value. The variables used were age (over 40 years old), weight (less than 60 kg), CD4 count (less than 500/μL), and the number of *6 and *28 alleles; they were analyzed using the forced input method. The cut-off value for age was set to 40 years old to match that of a previously published study [8]. To search for factors associated with selected NP-AEs, the cumulative incidence of NP-AEs was estimated by the Kaplan-Meier method. The subjects were divided into two groups, stratified by the presence or absence of carrying UGT1A1*6, UGT1A1*28, or both alleles (normal allele group, −/− versus reduced-function allele group, −/*28, −/*6, *28/*28, *6/28, and *6/*6), and these two groups were compared. We adopted a significance level of 5%, and the statistical analysis was conducted using JMP Software version 10.0.0 (SAS Institute Inc., Cary, NC). The *p* value of χ^2^ in the analyses of Hardy-Weinberg equilibrium was calculated using R software, version 3.3.3 (R Foundation for Statistical Computing, Vienna, Austria).

## Results

### Frequency of UGT1A1 gene polymorphisms in Japanese patients with HIV-1 infection

Table 1 shows the general characteristics of the 107 individuals infected with HIV-1 who participated in this study based on the type of *UGT1A1* gene polymorphism. The subjects comprised eight (7%) *6 homozygotes, three (3%) *28 homozygotes, four (4%) *6/*28 compound heterozygotes, 23 (21%) *6 heterozygotes, 18 (17%) *28 heterozygotes, and 51 (48%) patients homozygous for the wildtype *UGT1A1* genotype. The *6 and *28 allele frequencies were 20% and 13%, respectively. Distribution of the *6 and *28 allele was in the Hardy-Weinberg equilibrium (*p* = 0.83 and *p* = 0.92, respectively). Table 1 shows that there were no significant differences among *UGT1A1* gene polymorphism frequencies with respect to the listed participant characteristics, except for rilpivirine use that was due to one individual.

**Table 1:** Demographics and genotypes of participants

Genotype	−/−	−/*28	−/*6	*28/*28	*6/*28	*6/*6	*p*-value
Participants (n, %)	51 (48%)	18 (17%)	23 (21%)	3 (3%)	4 (4%)	8 (7%)	
Age (year), median [IQR]	44 [39–51]	43 [39–53]	42 [38–45]	42 [41–43]	46 [44–51]	44 [38–50]	0.8351
Males (n, %)	48 (94%)	15 (83%)	20 (87%)	3 (100%)	4 (100%)	8 (100%)	0.5445
Body weight (kg), median [IQR]	65 [58–72]	66 [58–72]	67 [61–74]	64 [61–77]	55 [51–59]	68 [60–73]	0.4805
Treatment naïve (n, %)	9 (18%)	5 (28%)	9 (39%)	0 (0%)	1 (25%)	2 (25%)	0.4084
CD4 cell count (cells/mL), median at the time of sampling [IQR]	486 [329–616]	431 [366–552]	378 [331–546]	434 [406–667]	500 [405–628]	540 [485–583]	0.5838
Participants with <50 HIV-1-RNA levels at the time of sampling (n, %)	50 (98%)	18 (100%)	22 (96%)	3 (100%)	4 (100%)	8 (100%)	0.9241
CD4 cell count (cells/mL), median at 24 weeks [IQR]	511 [336–603]	464 [402–623]	424 [335–528]	638 [507–819]	464 [402–563]	642 [584–669]	0.1145
Participants with <50 HIV-1-RNA levels at 24 weeks (n, %)	51 (100%)	18 (100%)	23 (100%)	3 (100%)	4 (100%)	8 (100%)	1.000
Use of antiretroviral agents (n, %)							
Tenofovir disoproxil fumarate	26 (51%)	9 (50%)	12 (52%)	2 (67%)	4 (100%)	4 (57%)	0.5559
Abacavir sulfate	25 (49%)	9 (50%)	11 (48%)	1 (33%)	0 (0%)	3 (43%)	0.4298
Rilpivirine hydrochloride	0 (0%)	0 (0%)	0 (0%)	0 (0%)	0 (0%)	1 (13%)	0.0286
Duration of DTG treatment (days), median [IQR]	111 [63–169]	112 [78–215]	77 [60–117]	67 [62–111]	67 [44–113]	68 [30–94]	0.3474
Food consumption							0.2698
Fasting	16 (31%)	9 (50%)	8 (35%)	1 (33%)	3 (75%)	5 (63%)	
Light meal	35 (69%)	9 (50%)	15 (65%)	2 (67%)	1 (25%)	3 (37%)	
HBV infection (n, %)	4 (8%)	1 (6%)	0 (0%)	0 (0%)	0 (0%)	1 (13%)	0.7028
HCV infection (n, %)	2 (4%)	0 (0%)	0 (0%)	0 (0%)	0 (0%)	0 (0%)	1.0000

*IQR* interquartile range, *HIV* human immunodeficiency virus, *DTG* dolutegravir, *HBV* hepatitis B virus, *HCV* hepatitis C virus

### Relationship between UGT1A1 gene polymorphisms and plasma DTG concentrations

**Fig. 1:**
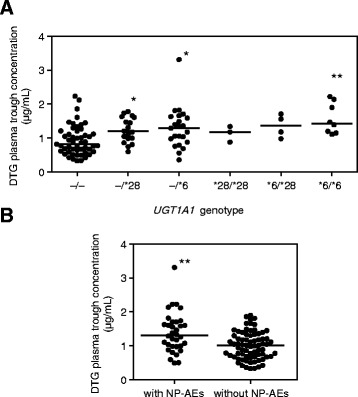
**a** Correlation between uridine diphosphate-glucuronosyltransferase 1A1 (*UGT1A1*) polymorphisms and plasma dolutegravir (DTG) trough concentrations. The horizontal straight lines indicate the median values. The Wilcoxon rank-sum test revealed significant differences between the groups (*p* = 0.0008). Post-hoc testing was performed using the Steel’s test; a comparison was made to the normal allele group. * *p* < 0.05 and ** *p* < 0.01. **b** Plasma DTG trough concentrations with or without neuropsychiatric adverse events (NP-AEs) after the use of DTG. The Wilcoxon rank-sum test showed significant differences (** *p* = 0.0013)

The median DTG trough concentration of the 107 subjects was 1.06 μg/mL. First, the DTG trough concentrations were classified based on *UGT1A1* gene polymorphisms and group comparisons were conducted (Fig. 1a, Wilcoxon rank-sum test, *p* = 0.0008). The median DTG trough concentration of the *6 homozygous patients (1.43 μg/mL) was significantly higher than that of patients carrying both normal alleles (0.82 μg/mL, *p* = 0.0054). The median concentrations of the *6 and *28 heterozygous patients (1.29 and 1.2 μg/mL, respectively) were also significantly higher than that of the homozygous patients carrying normal alleles (*p* = 0.0223 and *p* = 0.0254, respectively). Moreover, no statistically significant difference was detected in median DTG trough concentrations between patients with both normal alleles and those with both *28 alleles (1.18 μg/mL) or between those with both normal alleles and *6/*28 compound heterozygotes (1.37 μg/mL). However, we could not rule out the possibility that the statistical power was insufficient due to the small numbers of patients.

Next, a multivariate logistic regression analysis was conducted using the median DTG trough concentration, 1.06 μg/mL, as the cut-off value to find factors associated with high DTG trough concentrations. Carrying one or two UGT1A1*6 gene alleles and a UGT1A1*28 allele, as well as an age < 40 years were found to be independent factors associated with high DTG trough concentrations (Table 2).

**Table 2:** Associations between parameters and high dolutegravir (DTG) plasma trough concentrations (≥ 1.06 μg/mL)

	Univariate results			Multivariate results		
	OR	95% CI	*p*-value	OR	95% CI	*p*-value
Age ≥ 40 years	0.35	0.15–0.82	0.0144	0.35	0.13–0.92	0.0324
Body weight < 65 kg	0.71	0.33–1.53	0.3864	1.02	0.41–2.57	0.9705
Sex (Male)	3.95	0.90–27.5	0.0693	3.84	0.73–30.5	0.1175
HIV-1-RNA level at the time of sampling (< 50 copies/mL)	0.98	0.04–25.2	0.9894	0.53	0.02–15.7	0.6786
Number of UGT1A1*6 alleles						
0	1			1		
1	2.52	1.02–6.46	0.0434	3.33	1.24–9.51	0.0164
2	4.4 × 10^7^	5.21-∞	0.0003	5.9 × 10^7^	6.90-∞	0.0001
Number of UGT1A1*28 alleles						
0	1			1		
1	2.03	0.78–5.57	0.1476	3.61	1.24–11.5	0.0184
2	2.32	0.21–50.9	0.4862	4.01	0.33–93.4	0.2669

*OR* odds ratio, *CI* confidence interval, *UGT1A1* uridine diphosphate-glucuronosyltransferase 1A1, *HIV* human immunodeficiency virus

### Examination of the factors associated with NP-AEs

**Fig. 2:**
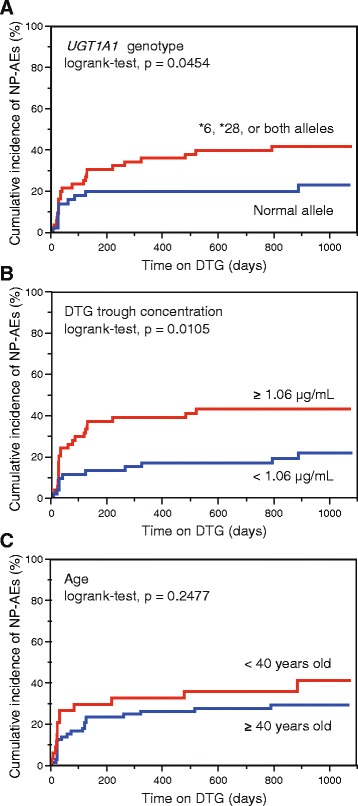
Kaplan-Meier estimation of the cumulative incidence of neuropsychiatric adverse events (NP-AEs). **a** *Blue*, and *red lines* indicate the Kaplan-Meier curves in the subjects carrying both normal alleles, and UGT1A1*6, UGT1A1*28, or both alleles, respectively. **b** Comparison between the subjects with lower (< 1.06 μg/mL, *blue line*) and higher (≥ 1.06 μg/mL, *red line*) plasma DTG trough concentrations. **c** Comparison between older (≥ 40 years old, *blue line*) and younger (*red line*) subjects

During the follow-up period (255 person-years), selected NP-AEs after the use of DTG were observed in 34 subjects (32%, Table 3). Insomnia was the most frequent (*n* = 14, 13%) among selected NP-AEs, followed by dizziness (*n* = 9, 8%), and headache (n = 9, 8%). The severity of all NP-AEs was grade 1 or 2. Six subjects discontinued DTG, and all were because of NP-AEs. Two subjects were lost to follow-up because of personal reasons. As shown in Fig. 1b, plasma DTG trough concentration was significantly higher in the patients with NP-AEs (median, 1.31 μg/mL) than in those without NP-AEs (median, 1.01 μg/mL, Wilcoxon rank-sum test, *p* = 0.0013). Next, we estimated the factors associated with the cumulative incidence of selected NP-AEs by use of Kaplan-Meier curves (Fig. 2). Plasma DTG trough concentration (≥ 1.06 μg/mL), and carrying UGT1A1*6, UGT1A1*28, or both alleles were associated with higher cumulative incidences of selected NP-AEs (log rank-test, *p* = 0.0105, and 0.0454, respectively). Age did not show this association (log rank-test, *p* = 0.2477).

**Table 3:** Selected neuropsychiatric adverse events (NP-AEs) after the use of dolutegravir (DTG) among the uridine diphosphate-glucuronosyltransferase 1A1 (*UGT1A1*) genotypes

	−/−	−/*28	−/*6	*28/*28	*6/*28	*6/*6
	*n* = 51	*n* = 18	*n* = 23	*n* = 3	*n* = 4	*n* = 8
Insomnia (n)	4	4	2	1	1	2
Headache (n)	2	2	3	1	0	1
Dizziness (n)	5	3	0	0	1	0
Restlessness (n)	2	0	1	0	1	0
Anxiety (n)	0	0	1	0	1	0
At least one NP-AEs (n, %)	11 (22%)	8 (44%)	7 (30%)	2 (67%)	3 (75%)	3 (38%)

## Discussion

The rate of carriers with the *UGT1A1* gene polymorphism has been reported to vary depending on race. The allele frequency of the *28 homozygote has been reported to be approximately 30–40% in Caucasian and African American populations [10], and 10–13% in Japanese populations [10, 12]. In contrast, the *6 homozygote is rarely detected in Caucasian and African American populations but is specifically found in Asian populations with a frequency of approximately 16% [12]. Similar allele frequencies were found in this study, and, therefore, the subjects are unlikely to deviate from a general Japanese population.

In this study, the median DTG trough concentration of the *6 homozygous patients was approximately 1.7 times higher than that of the subjects carrying both normal alleles. On the other hand, in our previous study on RAL, the median RAL trough concentration of *6 homozygous patients was approximately 10 times higher than that of patients carrying both normal alleles [21]. These observations indicate that the UGT1A1*6 had a smaller effect on the DTG trough concentration than it did on the RAL trough concentration. However, this interpretation is limited by the small number of *6 homozygous patients in both studies. Possible factors for the differential effects include a difference in the mediating metabolic pathway between RAL and DTG. RAL is predominantly metabolized by the UGT1A1 pathway and is not a substrate of CYP enzymes. In contrast, the principal pathway responsible for metabolizing approximately 70% of DTG involves UGT1A1, while CYP3A and other enzymes are involved in the secondary metabolic pathway of the remaining 30% [23]. This secondary metabolic pathway could reduce the effect of UGT1A1*6 on the plasma DTG concentration.

Unexpectedly, age < 40 years was an independent factor associated with high DTG trough concentrations. Drug blood concentrations increase with age. However, a population pharmacokinetic analysis in a previous study also showed the association between older age and decreased DTG plasma trough concentration [8]. Several factors could be considered responsible for this observation, including reduced absorption of DTG in the gastrointestinal tract, increased clearance of DTG due to low levels of plasma albumin resulting in an increased unbound fraction of plasma DTG [8, 24], and altered body fat, which affects the distribution of highly lipid-soluble drugs [25]. However, these reasons seem to be unlikely in this study because these physiological changes are apparent in the elderly [25, 26], and 93% of the subjects in this study were 20–50 years old. Further study is required to clarify the reason for the age effect.

This study demonstrated that plasma DTG trough concentrations were higher in the cases with NP-AEs than in those without NP-AEs. In addition, an association between *UGT1A1* gene polymorphism and NP-AEs was suggested. The mechanism of the latter seems to be that reduced-function alleles of *UGT1A1* can induce NP-AEs by increasing plasma DTG concentrations. In addition, *UGT1A1* gene polymorphism, age, gender, body weight, smoking, and food consumption were reported to affect the pharmacokinetics of DTG [8]. Therefore, it seems reasonable that plasma DTG trough concentration showed a stronger association with NP-AEs than *UGT1A1* gene polymorphism. Similar to the effect of DTG in this study, plasma efavirenz (EFV) concentration was reported to be associated with toxicities of the central nervous system [27]. That study suggested that a 1–4 μg/mL range at mid-dosing interval was a suitable target for plasma EFV concentration. However, it was difficult to set a cut-off value for DTG in our study, because we did not include patients who stopped taking DTG within 150 days of initiation. In a retrospective study about cases of DTG discontinuation, the median DTG trough concentration of 12 cases was reported to be 1.72 μg/mL [20]. Considering these observations, more detailed pharmacokinetics about the DTG discontinuation cases may be required to set appropriate cut-off values to predict the risk of NP-AEs after the use of DTG.

This study has some limitations. For example, this was an observational study with a limited number of patients at a single center. Especially, the number of *6 and *28 homozygous and *6/*28 compound heterozygous patients was small. The plasma DTG concentration was evaluated only using trough concentration. Furthermore, our cohort may not be representative of the entire population and the discontinuation rate of DTG in this study may differ from that of a typical setting in Japan. Possible reasons for these differences include that we could not recruit patients who stopped taking DTG before plasma DTG concentrations reached steady state, and that the subjects were selected without use of random sampling techniques. Recently, it was reported that gene polymorphisms of drug transporters influenced plasma RAL peak concentrations [28]. It is likely that gene polymorphisms of drug transporters can affect the pharmacokinetics of DTG as well. Gene polymorphisms CYP3A4*22 and CYP3A5*3 could also alter the pharmacokinetics of DTG, because DTG is partially metabolized by CYP3A and these polymorphisms were reported to change the metabolism of tacrolimus [29]. Further studies should be conducted to examine the pharmacokinetics of DTG and the associated factors in addition to UGT.

## Conclusions

We demonstrated that carrying UGT1A1*6, UGT1A1*28, or both alleles, as well as younger age, were factors resulting in high trough concentrations of DTG. In addition, our results suggest that a relationship exists between plasma DTG trough concentrations and the development of selected NP-AEs after the use of DTG. Carrying UGT1A1*6 and/or UGT1A1*28 alleles may also be a risk of developing NP-AEs.

## Abbreviations

CYP3A: Cytochrome P450 3A
DTG: Dolutegravir
HIV: Human immunodeficiency virus
NP-AEs: Neuropsychiatric adverse events
RAL: Raltegravir
UGT1A1: Uridine diphosphate (UDP)-glucuronosyltransferase 1A1
